# Ultrasensitive and real-time optical detection of cellular oxidative stress using graphene-covered tunable plasmonic interfaces

**DOI:** 10.1186/s40580-022-00315-9

**Published:** 2022-05-23

**Authors:** Hakchun Kim, Hyun Ji An, Junhee Park, Yohan Lee, Min Seob Kim, Seungki Lee, Nam Dong Kim, Jihwan Song, Inhee Choi

**Affiliations:** 1 Department of Life Science, University of Seoul, Seoul, 02054 Republic of Korea; 2 Department of Mechanical Engineering, Hanbat National University, Daejeon, 34158 Republic of Korea; 3 Institute of Advanced Composite Materials, Korea Institute of Science and Technology, Bongdong-eup, Wanju-gun, Jeollabuk-do 55324 Republic of Korea

**Keywords:** Graphene, Oxidative stress, Plasmonic nanoparticles, Plasmonic resonance energy transfer, Reactive oxygen species

## Abstract

Reactive oxygen species (ROS) regulate various physiological and pathological conditions in cells by interacting with signaling molecules and inducing oxidative stress. Therefore, sensitive monitoring of ROS levels in living cells is important to track cellular state and study the complex role of ROS in the development of various pathologies. Herein, we present an optically tunable plasmonic interface covered with graphene to monitor cellular ROS levels with superior sensitivity and cellular comfortability. As a sensing principle, we employed plasmon resonance energy transfer (PRET)-based spectral quenching dips modulated by redox-active cytochrome *c* for real-time monitoring. By transferring graphene layers to plasmonic nanoparticles immobilized on a glass substrate, the scattering profiles of the nanoprobes were adjusted in terms of the position, width, and intensity of the peaks to determine the optimal conditions for measuring the PRET signal. Using the optimized graphene-covered plasmonic nanoprobe, we obtained calibration curves over a wide concentration range from femtomoles to millimoles for hydrogen peroxide based on the change in the PRET signal. Before monitoring cellular ROS, we confirmed that a high density of cells adhered well to the graphene-covered plasmonic interface by observing immunofluorescence images of the cytoskeleton of the immobilized cells. Finally, we monitored the real-time ROS generated by the cells under oxidative stress conditions by directly measuring the spectral changes of the probes around the cells. We believe that the proposed graphene-covered tunable plasmonic interface has versatile applicability for investigating cellular stress and disease progression by monitoring ROS levels under various cellular conditions.

**Supplementary information:**

The online version contains supplementary material available at 10.1186/s40580-022-00315-9.

## Introduction

Reactive oxygen species (ROS), like superoxide anions, hydrogen peroxide, and hydroxyl radicals, are molecules with strong oxidative power and high reactivity that regulate a variety of biological processes [1–4]. In normal oxygen consumption and cellular aerobic metabolism, generally by mitochondrial respiration, some of the oxygen entering the mitochondria is not completely oxidized, resulting in the generation of ROS as a byproduct [1, 4, 5]. The generated ROS are directly involved in the physiological regulation of cellular signaling pathways and oxidative stress [1, 6–9]. ROS affect cell proliferation [10, 11], differentiation [11, 12], DNA damage [12, 13], and apoptosis [14, 15] through signaling processes. Low levels of ROS production can enhance the proliferation [4, 11, 13, 16] of cells, whereas their excessive levels lead to cell necroptosis by oxidative damage to DNA [9, 17], lipids [3, 18], and proteins [10, 16]. As ROS play important roles in determining cellular lifespans, it is important to sensitively detect their levels in cells [10, 12–14, 19–22].

Various methods have been developed to detect ROS generated by cells using fluorescence [23–27], chemiluminescence [28–30], and chromatography [31, 32]. However, most conventional methods have limitations in real-time monitoring of ROS generated from living cells owing to the requirement of sample pretreatment steps and reaction times with reagents. To date, there have been limited reports on the real-time optical monitoring of ROS. For example, the fluorescence method using redox-sensitive green fluorescent protein (GFP) allows real-time visualization of the oxidation state of GFP induced by ROS [33, 34]. However, the low sensitivity and photobleaching of fluorescence probes remain challenging issues for achieving long-term monitoring of cellular ROS. To overcome these drawbacks, plasmonic nanoparticles, such as silver nanoparticles (SNP) and gold nanoparticles (GNP), have been used in sensing [35, 36] and cellular imaging [37, 38], owing to their excellent optical properties, photostability, and easy surface functionalization. Using these plasmonic nanoparticles, a new principle for ROS monitoring based on plasmon resonance energy transfer (PRET) has recently been demonstrated [39, 40]. In brief, PRET from plasmonic nanoprobes to redox-active cytochrome *c* (Cyt *c*) induces unique spectral quenching dips in the scattering profile of the plasmonic probe, which can be changed by the presence of ROS. Before cellular ROS monitoring, it would be better to use optically adjustable and biocompatible interfaces for monitoring oxidative stress in cells. Because the PRET signals stem from the spectral overlap between the scattering of the plasmonic nanoprobe and the absorption of the redox-active Cyt *c*, fine-tuning of the scattering spectra of the probes is essential to achieve high sensitivity. Moreover, a comfortable interface should be provided to cells to monitor the oxidative stress experienced under certain extracellular or intracellular conditions. Recent reports have shown that diverse cell types, including fibroblasts [41], neurons [42], and osteoblasts [43], adhere well and proliferate on graphene, suggesting that the graphene layer serves as a comfortable cellular interface.

**Fig. 1:**
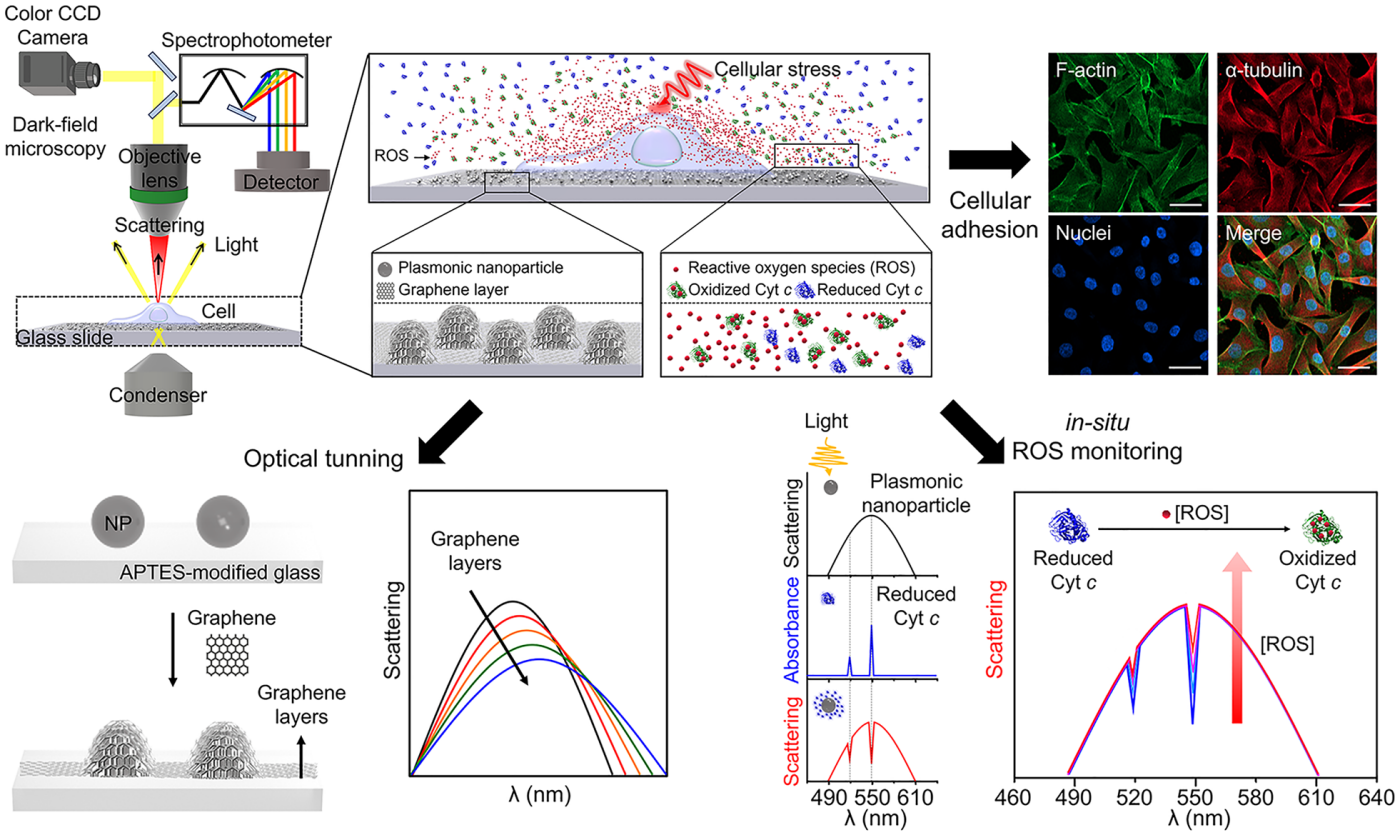
Overall schematic illustration of ROS monitoring generated from living cells using plasmonic NP-graphene interface. (Upper left) Hyperspectral imaging setup for in-situ monitoring cellular ROS on the graphene-covered plasmonic nanoprobes. (Upper right) Representative images of the cells adhered to the graphene-covered plasmonic nanoprobes. (Lower left) Schematic illustration showing the graphene transfer process on plasmonic nanoparticles pre-immobilized on the APTES-modified glass substrate and optical tunability according to the graphene layers. (Lower right) Principle of ROS monitoring through the measurement of changes in quenching dip at 550 nm for redox-active Cyt *c*

In this study, we demonstrate a plasmonic interface covered with graphene layers to optically monitor the ROS generated from living cells. As illustrated in Fig. 1, the graphene layers were transferred to plasmonic nanoparticles on a glass substrate as the cellular interface. Here, the graphene layer provides an effective optical interface for PRET measurements and simultaneously provides a chemically inert and comfortable surface for cells. Using this cellular interface, ROS levels were monitored via PRET signals, represented as spectral quenching dips, induced by the interaction between the single plasmonic nanoprobe and redox-active Cyt *c* reacted with ROS. By measuring PRET signals in real time, we can quantitatively detect ROS levels and monitor cellular ROS.

## Methods/experimental

### Materials

A poly(polydimethylsiloxane) (PDMS) elastomer kit (Sylgard 184) was purchased from Dow Corning (Midland, MI, USA). Copper (Cu) foil (0.025 mm thick, annealed, uncoated, 99.8%) was purchased from Alfa Aesar (Haverhill, MA, USA). Sulfuric acid (H_2_SO_4_, 95%), hydrogen peroxide (H_2_O_2_, 34.5%), ammonium persulfate (98%), ethanol (99.9%), dimethyl sulfoxide (99.8%), acetone (99.5%), 2-propanol (IPA, 99.5%), and nitric acid (69%) were purchased from Samchun Pure Chemical Co. Ltd. (Seoul, Korea). Silver nanoparticle (SNP, 100 nm), cytochrome *c* (Cyt *c*) from the equine heart (≥ 95%), l-ascorbic acid (AA), 3-aminopropyltriethoxysilane (APTES), phosphate-buffered saline (PBS), poly-l-lysine (PLL) solution (0.01%), sodium (meta) arsenite (≥ 90%), Triton X-100, bovine serum albumin (BSA), and Hoechst 33258 were purchased from Sigma-Aldrich (St. Louis, MO, USA). Gold nanoparticles (GNP, 50 nm) were purchased from BBI solutions (Cardiff, UK). The human dermal fibroblast cell line (HDF) was obtained from NeoRegen Biotech Co., Ltd. (Seoul, Korea). Dulbecco’s modified Eagle medium (DMEM), penicillin/streptomycin (Penstrep), and 0.05% trypsin-EDTA were purchased from Gibco-Life Technologies (Mulgrave, Australia). Fetal bovine serum (FBS) was purchased from MP Biomedicals (Irvine, CA). Formaldehyde (4%) and Alexa Fluor 488 phalloidin were purchased from Thermo Fisher Scientific (Waltham, MA, USA). Rabbit monoclonal anti-α-tubulin and Alexa 647-conjugated anti-rabbit secondary antibodies were purchased from Abcam (Cambridge, MA, USA).

### Optical simulation of graphene-covered plasmonic NPs

Wave optics simulations were conducted to predict the scattering properties of the plasmonic NP-graphene interface according to the types of plasmonic NPs and the number of graphene layers. The simulation domain was composed of metal NPs and graphene layers placed on glass and immersed in a liquid. Both SNPs and GNPs were simulated, and planar electromagnetic waves were irradiated vertically from the bottom to the top along the z-axis with a wavelength in the range of 300–800 nm. All simulations were performed using a commercial software (COMSOL Multiphysics, 5.4; COMSOL, Inc.).

### CVD graphene growth

Before graphene growth, the Cu foils were cut into 10 cm × 10 cm pieces. Nitric acid was diluted in deionized (DI) water under stirring. The Cu foils were immersed in dilute nitric acid for approximately 30 s. The cleaned Cu foils were successively washed with isopropanol, acetone, and DI water three times. Monolayer graphene was then grown on the cleaned copper foil via a CVD system using CH_4_ and H_2_ as precursor gases. The substrate was first annealed at 1000 ℃ for 30 min under an H_2_ flow (100 sccm). Subsequently, the carbon source gas, CH_4_ (10 sccm), was introduced into the quartz tube. After 30 min of growth, the CH_4_ gas was turned off, and the copper substrate was removed from the heating area of the furnace to cool down at room temperature under H_2_ flow.

### Transmission electron microscopy (TEM)

TEM analysis was performed to characterize plasmonic NPs. To prepare the specimens, 10 µL of each NP solution was dropped onto a carbon-coated 300-mesh TEM grid (Ted Pella, Inc.). TEM images were obtained using a transmission electron microscope (LIBRA 120; Carl Zeiss, Jena, Germany) operating at an acceleration voltage of 120 kV.

### Fabrication of plasmonic NP-graphene interface

The glass slide was treated with piranha solution (H_2_SO_4_/H_2_O_2_ = 7:3 v/v) for 1 h, rinsed with ethanol and DI water, and then dried in a stream of N_2_. The cleaned glass slide was immersed in an ethanol solution of 1 mM APTES for 24 h. The surface modified with APTES was washed with pure ethanol and dried in a stream of N_2_. To immobilize plasmonic NPs on the glass slide, 200 µL of colloidal NP solution was dropped onto the APTES-modified glass slide for 30 s and washed with DI water to remove excess NPs. Prior to transferring the graphene layers onto the NP-immobilized glass substrate, the graphene was floated in a 1 M ammonium persulfate aqueous solution for 6 h to completely dissolve the Cu foil. Then, it was washed twice with DI water for 15 min and washed with HCl for 10 min at room temperature (RT) for complete etching of Cu. After the etched graphene was placed on a glass slide, the PMMA layer was removed with acetone and IPA.

### Scanning electron microscopy (SEM)

The surface morphology of the graphene-covered plasmonic NP was characterized using field-emission scanning electron microscopy (SU8010, HITACHI, Japan) at an accelerating voltage of 10 kV.

### Atomic force microscopy (AFM)

The surface topologies and height profiles of the transferred graphene layers were also obtained using an AFM (NX12-bio, Park Systems, Korea) in the non-contact mode.

### Raman measurement

A micro-Raman system combined with a spectrometer SR-303i (Andor Technology) and a 50 mW 532-nm laser module PSU-III-FDA (Changchun New Industries Optoelectronics Technology Co., Ltd.) was used for the characterization of graphene. The system was comprised of an integral Olympus BX51 microscope with a 20× objective lens. The Raman spectra were collected at an exposure time of 1 s (five accumulations).

### Dark-field scattering imaging and spectral analysis

Dark-field scattering imaging of graphene-covered plasmonic NPs according to the number of graphene layers and the corresponding spectral analysis under each condition were performed using a dark-field microscope (Olympus BX43, Tokyo, Japan) equipped with a hyperspectral imaging spectrophotometer (CytoViva, Auburn, AL, USA). A 20× objective lens was used for imaging, and the integration time for collecting the scattering spectra was 0.3 s. For all data, the reliabilities of the measured intensity, spectral shifts, and full width at half maximum (FWHM) were evaluated based on the standard deviation from 60 different nanoprobes.

### Photoluminescence measurement

The photoluminescence (PL) properties of the plasmonic NP-graphene interface were characterized using a fluorescence spectrometer (FlouTime 300, PicoQuant, Berlin, Germany) with an excitation picosecond laser at 520 nm in the range 520–900 nm.

### PRET-based ROS detection

To demonstrate PRET-based ROS quantification, changes in the quenching dips of the single plasmonic NPs depending on the H_2_O_2_ concentration were measured using the above-mentioned dark-field-based hyperspectral system. Prior to the measurement of the quenching dips, the intrinsic spectra of the single NPs were measured under DI water in the PDMS well. To measure the initial spectra of the NPs before exposure to H_2_O_2,_ DI water in the reaction well was replaced with a reduced Cyt *c* aqueous solution as a redox probe, which was prepared by preincubation with 100 µM Cyt *c* and 50 mM AA (5:1 v/v) for 1 h. Then, H_2_O_2_ was injected into the PDMS well, and the ratio between the reduced Cyt *c* and H_2_O_2_ was set to 5:1 v/v. After a 2 min reaction, the scattering spectra of the single NPs because of H_2_O_2_ exposure were collected. All collected spectra were normalized, and changes in the spectral quenching dips were measured before and after H_2_O_2_ exposure. For all the data, the reliability of the observed spectral shift and intensity was examined using the standard deviation of 60 different NPs.

### Immunofluorescence staining

To examine the biocompatibility of the plasmonic NP-graphene interface, cells incubated for 24 h were fixed with 4% formaldehyde in PBS for 15 min at RT and permeabilized with 0.4% Triton X-100 for 15 min. The cells were washed twice with PBS for 15 min. For imaging microtubules, after blocking the cells with 5% BSA in PBS for 1 h, cells were incubated with a rabbit monoclonal anti-α-tubulin antibody (EP1332Y; Abcam, Cambridge, MA, USA) overnight at 4 ℃ and washed five times with PBS for 2 min over 5 times. The cells were further incubated with an Alexa 647-conjugated anti-rabbit secondary antibody (ab150083; Abcam, Cambridge, MA, USA) for 1 h at RT. F-actin was stained with Alexa Fluor 488-phalloidin (Invitrogen) for 1 h at RT. Nucleic acids were stained with the DNA-binding dye Hoechst 33258 (94403; Sigma-Aldrich). Stained cells were visualized using a confocal laser scanning microscope (LSM 800; Carl Zeiss, Jena, Germany).

### Real-time monitoring of ROS generated from living cells

HDF cells (5 × 10^3^ cells/200 µL) and A375P (5 × 10^3^ cells/200 µL) were seeded in 0.01% PLL-treated plasmonic NP-graphene interface and incubated at 37 °C and 5% CO_2_ atmosphere for 24 h. Then, 0.2 M NaAsO_2_ and reduced Cyt *c* diluted in DMEM were injected onto the plasmonic NP-graphene interface in the PDMS well. The change in the scattering spectra induced by the ROS was monitored using a dark-field-based hyperspectral system.

### Fluorescence detection of intracellular ROS

Cells were seeded at 0.01% PLL-treated plasmonic NP-graphene interface and incubated at 37 ℃ in a 5% CO_2_ atmosphere for 24 h. To detect intracellular ROS under oxidative stress, cells were exposed to DMEM containing toxicants (e.g., 0.2 M NaAsO_2_). After incubation with the toxicants for 1 h, the cells were washed with PBS and exposed to 20 µM 2,7-dichlorofluoroscein diacetate (DCFDA) diluted in DMEM. After incubation for 50 min at 37 °C in the dark, the cells were washed with PBS and imaged using a confocal laser-scanning microscope.

## Results and discussion

### Predicting scattering properties of the plasmonic NPs covered with graphene layers

**Fig. 2:**
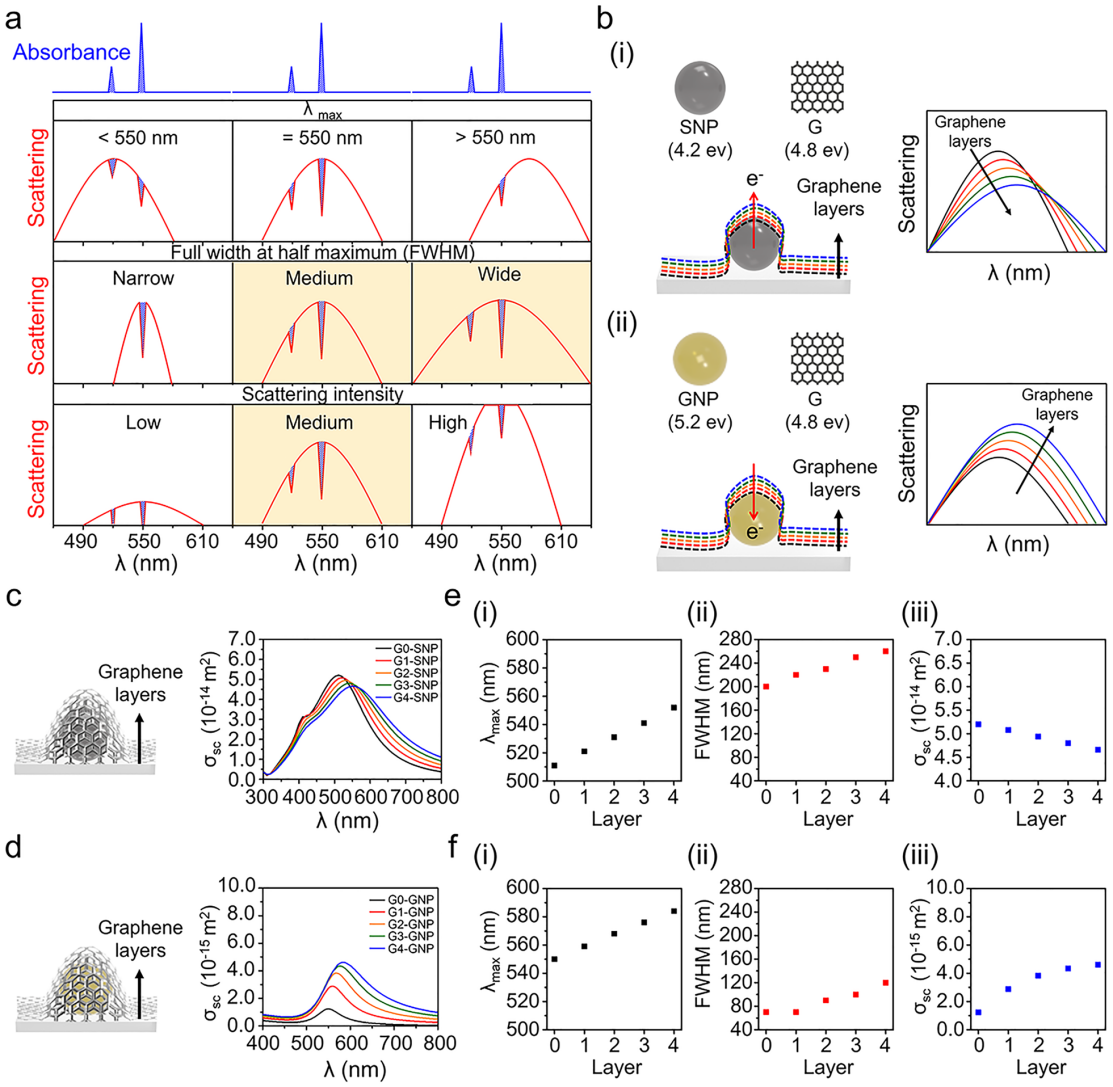
Cyt *c*-mediated PRET signals according to scattering profiles of nanoprobes and optical tuning strategy of the nanoprobes with graphene layers. **a** Expected features of the Cyt *c*-mediated PRET signal according to scattering profiles of nanoprobes in terms of λ_max_, FWHM, and intensity. The cases where two unique quenching dips for Cyt *c* were clearly observed are highlighted with yellow boxes. **b** The schematic diagram for the relationship of the electron movement through the NP-graphene interface with the change in scattering property according to the covered graphene layers, (i) SNP (ii) GNP. **c**, **d** Scattering spectra numerically calculated for the graphene-covered SNP (**c**) and GNP (**d**) with increasing number of graphene layers. In Gn-SNP and Gn-GNP, n indicates the number of graphene layers on the NPs. **e**, **f** Plots for the changes in scattering properties of SNP (**e**) and GNP (**f**) with increasing number of graphene layers, (i) λ_max_, (ii) FWHM, and (iii) scattering cross-section (σ_sc_)

In principle, PRET signals are measured in the form of spectral quenching dips in the scattering spectrum of the plasmonic nanoprobe matching with the molecular absorption bands when the light-absorbing molecules (Cyt *c* in this study) are near the plasmonic nanoprobes. That is, the PRET signal is a result of light energy transfer from plasmonic nanoparticles (NP) to light-absorbing molecules [44–47]. We note a unique absorption band at 520 and 550 nm for reduced Cyt *c.* As summarized in Fig. 2a. In the highlighted cases in Fig. 2a, two unique quenching dips in the absorption band for Cyt *c* were clearly observed without omitting or truncating the dips. This revealed that there is an optimal condition for measuring the Cyt *c*-mediated PRET signal in terms of the position, width, and intensity of the scattering peaks of the plasmonic nanoprobes. When we layer the optically transparent and ultra-thin graphene—an excellent electrical conductor—onto plasmonic nanoprobes, we expected that electron transfer between them can modulate the scattering properties of the probes according to the type of plasmonic NPs, as shown in Fig. 2b.

Before the experiment, we investigated the scattering behavior of the plasmonic NP-graphene interface through computational simulations. The simulations were performed using commercial software (COMSOL Multiphysics, 5.4; COMSOL, Inc.). The scattering cross-section (σ_sc_) of the nanoparticles was calculated by varying the sizes of the SNP and GNP and the number of graphene layers. For example, diameters of 100, 110, and 120 nm were considered for SNP, and diameters of 50, 55, and 60 nm were considered for GNP. In the case of graphene layers, 0–4 layers were considered (see Additional file 1: Figs. S1, S2). The thickness of a single graphene layer was set to 1 nm based on the experimentally reported value [48]. Figure 2c and d show the simulated scattering spectra of the 100 nm SNP and 50 nm GNP when they are covered with a different number of graphene layers. When the number of graphene layers increased, the scattering peak of the SNP red-shifted with increasing bandwidth and σ_sc_ decreased (Fig. 2e). In the case of GNP, the scattering peak shows a redshift with increasing bandwidth and σ_sc_ (Fig. 2f). For both SNP and GNP, the scattering peaks and bandwidth changes show similar trends with an increase in the number of graphene layers. In contrast, σ_sc_ showed the opposite trend for SNP and GNP. The σ_sc_ of SNP decreased with the increasing number of graphene layers, whereas that of GNP increased. This is due to their different refractive indices that are proportional to their work functions [49, 50]. Notably, we can predict that the efficiency of SNP-based PRET would be improved because the scattering peak moves to the vicinity of the reduced Cyt *c* absorption peak with a decrease in σ_sc_ when the graphene layer is present, although SNP shows a stronger σ_sc_ than GNP.

### Morphological properties of plasmonic NP-graphene interface

**Fig. 3:**
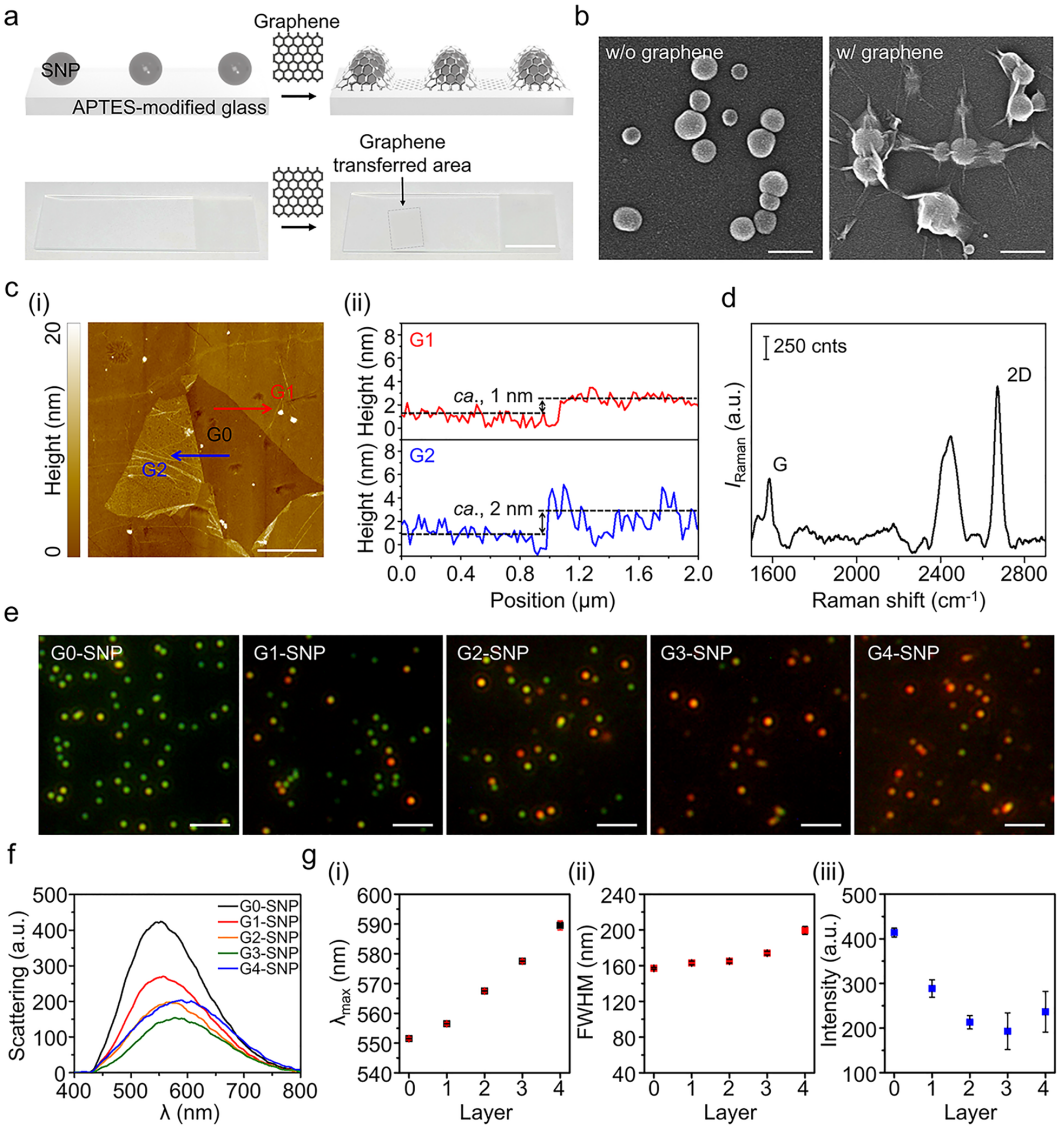
Characteristics of the graphene-covered plasmonic NPs and their optical properties. **a** Graphene transfer process to the NP immobilized on the APTES-modified glass substrate (upper) and photographs of substrates before and after transferring a graphene layer (lower). Scale bars represent 2 cm. **b** Scanning electron micrographs of the SNPs (left) and the graphene-covered SNPs (right). Scale bars represent 200 nm. **c** Topographic images (i) and height profiles (ii) of the graphene layers transferred on the glass substrate. Scale bar represents 2 μm. Height profiles correspond to the lines denoted as G1 and G2. **d** Raman spectra of the graphene-covered SNP substrate. **e** Dark-field scattering images of the graphene-covered plasmonic SNPs with increasing number of graphene layers. The scale bars represent 10 μm. **f** Corresponding scattering spectra measured for the SNPs with increasing number of graphene layers. **g** Plots for the shifts in terms of λ_max_ (i), FWHM (ii), and intensity (iii) with increasing number of graphene layers on the SNP

To prepare the plasmonic NP-graphene interface, plasmonic NPs were first immobilized on an amine-functionalized glass substrate, followed by transferring the graphene layer onto the immobilized NPs, as illustrated in Fig. 3a. Based on the simulation results, two types of plasmonic NPs—ca. 100 nm SNPs and 50 nm GNP—were used as optical probes as the position of their scattering bands matched with the absorption band of Cyt *c*. Based on TEM images, the average size of the used SNPs was 101.6 ± 5.0 nm (Additional file 1: Fig. S3). The average size of the GNPs was 49.5 ± 2.6 nm (Additional file 1: Fig. S4). In Fig. 3b, the SEM image shows a representative surface morphology of the plasmonic NP-graphene hybrid interface, indicating that the graphene layer covers the SNPs along the curvatures of the nanoparticles. To identify the quality of the transferred single graphene layer, its topographic image and height profile were obtained using AFM. In the topography, mono-layered and bi-layered graphene (induced by folding) were observed on the substrate (Fig. 3c(i)). As shown in Fig. 3c(ii), the height profiles clearly show the difference in the thickness of the monolayer graphene (*~* 1 nm) and bi-layered graphene (*~* 2 nm). Additionally, we measured the Raman scattering to further confirm the presence of the graphene layer in the plasmonic NP-graphene interface. As shown in Fig. 3d, the Raman spectrum of the fabricated interface presents vibrational peaks at 1585 cm^− 1^ (G peak) and 2670 cm^− 1^ (2D peak), which are well known as characteristic peaks of graphene [51, 52]. The peak intensity ratio of 2D to G was calculated as 2.34, which reflects the existence of a single graphene layer [48, 53]. These results indicated that the graphene layer was transferred onto the SNP probes.

### Tunable optical properties of plasmonic NPs covered with graphene layers

We next investigated the optical tunability of plasmonic NPs by increasing the number of graphene layers. Dark-field scattering images showed that the scattering colors of the SNP probes gradually changed from green to red as the number of graphene layers increased (Fig. 3e). Figure 3f shows the corresponding spectra of the individual SNP as the number of graphene layers increases as measured via dark-field microscopy combined with a spectrophotometer. A closer look at the spectral shift shows that the peak shifts from 551 to 589 nm, the bandwidth increases from 157 to 200 nm, and the intensity decreases by 42% when the number of graphene layers is four (Fig. 3g). The scattering properties of the GNPs covered with graphene layers were also tuned by varying the number of layers. The scattering color of the GNP probes changed from green to orange (Additional file 1: Fig. S5a). The scattering peaks red-shifted from 557 to 582 nm, increasing the bandwidth from 92 to 138 nm and increasing the intensity by 63% (for GNP covered with four layers) (Additional file 1: Fig. S5b, c). The trend of the spectral change according to the number of graphene layers in both SNP and GNP is consistent with the simulation results (see Fig. 2c, d). The discrepancy between simulation and experimental results in terms of numerical values can be attributed to the wider size distribution of the used NPs and thinner thickness of actual graphene layer than values used in the simulation. In addition, similar to the simulation results, the intensity of SNP was higher than that of GNP. In the case of the scattering intensity, the SNP showed a decreasing tendency with graphene layers, unlike the GNP (see Fig. 3f, g, and Additional file 1: Fig. S5b, c). As we predicted this based on simulation result, this is attributed to the different work functions of the interface materials. The work functions of Ag, Au, and graphene are 4.2, 5.2, and 4.8 eV (i.e., Ag < graphene < Au). When SNPs are in direct contact with graphene, free electrons would move from Ag to graphene in the SNP-graphene hybrid interface with the work function increasement due to the Fermi level shift effect [54–61]. On the other hand, electrons would move from graphene to Au and the work function would decrease in the GNP-graphene hybrid interface. To validate this, PL intensities of the NPs covered with a graphene layer were measured and compared with those of the uncovered NPs (Additional file 1: Fig. S6). The work function of a metal can be defined as the minimum energy required to extract one electron from a metal [62] and PL occurs when excited free electron is relaxed into a valence band. Therefore, it is known that metals with a low work function can easily extract electrons and increase the PL [63]. We observed that when incident light was illuminated to excite plasmon of the metallic surface, the PL intensity of the SNP-graphene hybrid interface decreased compared to that without graphene, and the PL intensity of the GNP-graphene hybrid interface increased compared to that without graphene, as shown in Additional file 1: Fig. S6. The change in the work function of the interface by covering the graphene layer induces a change in the PL intensity owing to electron transfer between the metallic NPs and graphene. As electrons move, energy is absorbed or emitted, affecting the scattering intensity. Thus, the scattering intensities were also tuned differently when Au and Ag were combined with graphene layers [62].

### Optimization of plasmonic NP-graphene interface for cyt *c*-mediated PRET

**Fig. 4:**
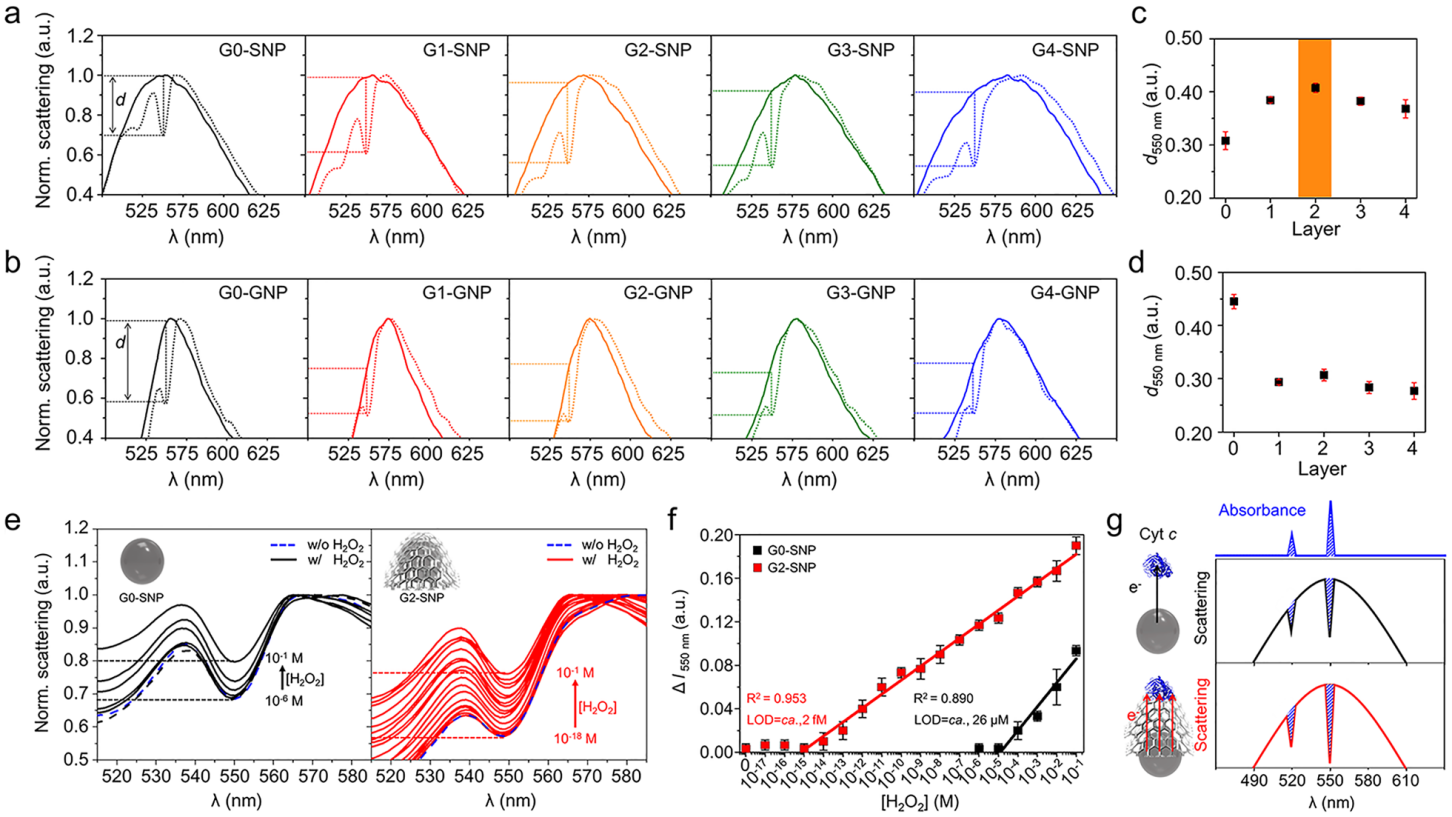
Optimization of the plasmonic NP-graphene interface for Cyt *c*-mediated PRET and ROS detection. **a**, **b** Cyt *c*-mediated PRET signals measured using SNP (**a**) and GNP (**b**) with increasing number of graphene layers. Solid lines and dotted lines indicate the probe spectra in the absence and presence of Cyt *c*, respectively. **c**, **d** Plots showing the change in depth of the quenching dip at 550 nm measured using SNP (**c**) and GNP (**d**) with increasing number of graphene layers. **e** H_2_O_2_ concentration-dependent changes in the spectral quenching dip of the SNP probe (left, G0-SNP) and SNP probe covered with two layers of graphene (right, G2-SNP). **f** Calibration curves for H_2_O_2_ obtained with G2-SNP (red) and G0-SNP (black). **g** Illustration showing the relationship between electron movement through the interface and the resulting PRET signal

To find an optimal plasmonic NP-graphene interface for sensitive ROS detection, the PRET signal from the reduced Cyt *c* was evaluated using the two types of plasmonic NPs (SNP and GNP) and their hybrid interfaces covered with different numbers of graphene layers. As expected, the quenching dips by PRET were the largest when the scattering peak of the nanoprobe exactly matched the absorption wavelength of Cyt *c* (see also Fig. 2a). The largest dip at 550 nm for the reduced Cyt *c* is profitable to sensitively monitor its change resulting from the oxidation of Cyt *c* by ROS. As shown in Fig. 4a, c, the depth of the quenching dip was the largest for the SNP covered with two layers of graphene. When using SNPs as probes, unique double quenching dips at 520 and 550 nm for the reduced Cyt *c* were clearly observed owing to the good spectral overlap between the scattering of the SNP and absorption of Cyt *c*. On the other hand, when GNPs were used as probes, the largest dip was observed for the GNP probe without a graphene layer, and a single quenching dip was observed only at 550 nm owing to the relatively narrow scattering band, and the quenching dip decreased with the increase in the number of graphene layers (Fig. 4b, d). As discussed earlier, electron movement from graphene to GNP [63, 64] would also result in decreased energy transfer to Cyt *c*. Taken together, the SNP covered with bilayer graphene was selected as the probe for Cyt *c*-mediated ROS detection.

For ROS detection, we intentionally prepared fully reduced Cyt *c*, whose absorption peaks exist at 520 and 550 nm, clearly distinguished from a single absorption peak around 530 nm of oxidized Cyt *c* [65]. ROS-induced oxidation of reduced Cyt *c* results in a change in the spectral quenching dip, which serves as a signal for ROS detection. Using two types of SNP probes, with and without graphene layers, we measured the PRET signals of Cyt *c* interacting with varying concentrations of H_2_O_2_, a representative ROS molecule. As shown in Fig. 4e, the position of the quenching dips proportionally increased with the concentration of H_2_O_2_, resulting from the H_2_O_2_-induced gradual oxidation of the reduced Cyt *c*. Based on the change in the quenching dip at 550 nm, we obtained a calibration curve for H_2_O_2_ over a wide concentration range from 10^− 1^ M to 10^− 18^ M (Fig. 4f). The SNP probe covered with bilayer graphene showed a good linear relationship on a logarithmic scale of H_2_O_2_ concentration over a wide concentration range from 10^− 1^ M to 10^− 15^ M, with a regression coefficient of 0.953. Based on the 3δ/slope method, the limit of detection (LOD) was calculated as 2 fM, which is six orders of magnitude lower than that of the commercially available ROS assay kit (ca. 1 µM) [59]. This is an ultrasensitive level and much lower than previously reported LOD values of the PRET-based detections conducted with Au-Pt nanoparticles (ca. 10 nM) [39] and Au-Pt cavities (ca. 1 nM) [40] without graphene layers. In contrast, the graphene-free SNP probe showed a regression coefficient of 0.890 and LOD of 26 µM. This result supports the importance of fine-tuning the scattering spectrum of the probe to achieve higher sensitivity in PRET-based detection, as we hypothesized in Fig. 2a. Furthermore, the achieved ultra-sensitivity can be attributed to the introduction of graphene layers to the SNP probe and the enhanced PRET efficiency to the increased electron transport from the SNP to Cyt *c*, as described in Fig. 4g.

### Real-time monitoring of ROS generated from living cells on the plasmonic SNP-graphene interface

**Fig. 5:**
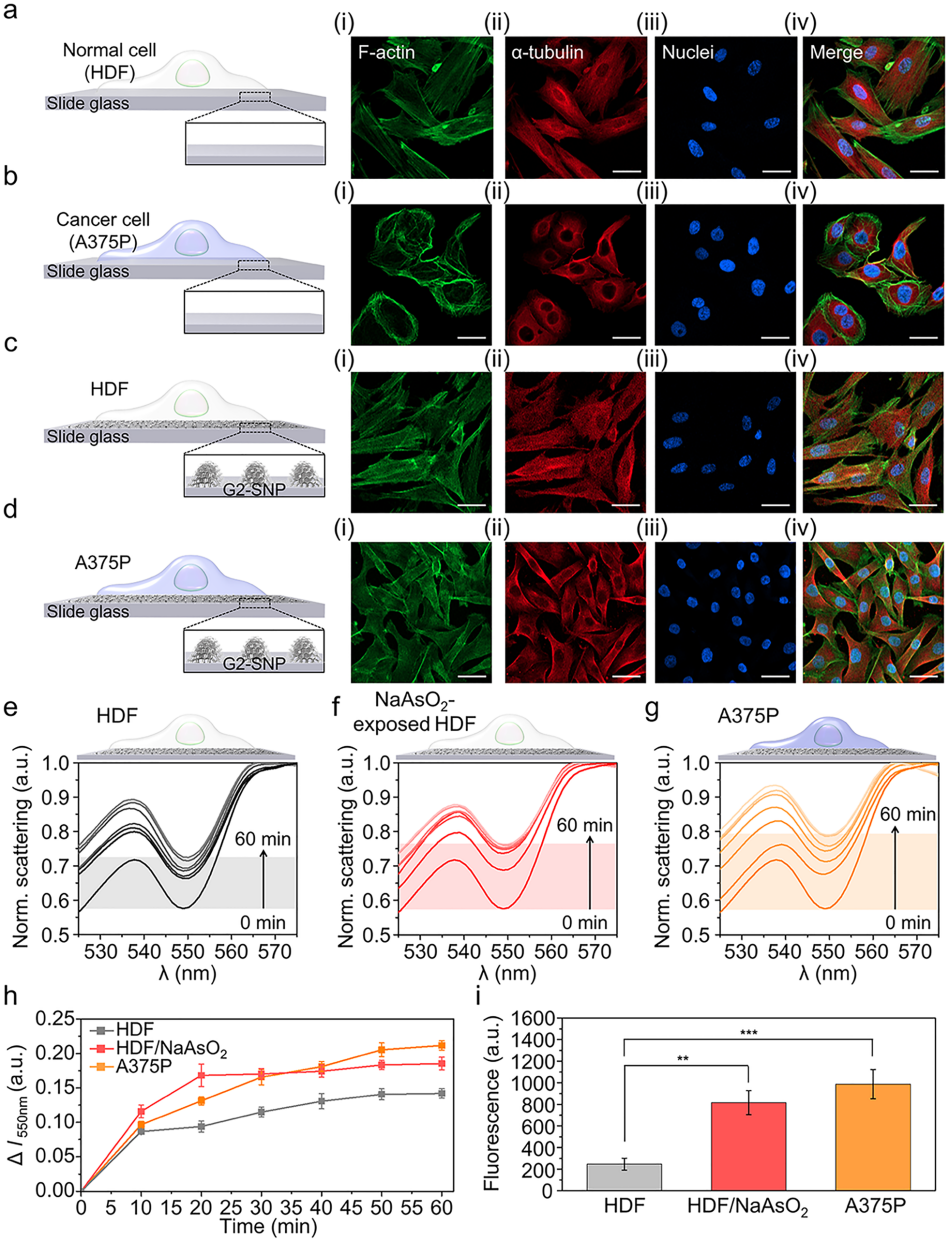
Real-time monitoring of ROS generated from the living cells on the plasmonic NP-graphene interface. **a**–**d** Immunofluorescence images of the normal cell (HDF) and cancer cell (A375P) cultured on the glass slide. **a**, **b**), plasmonic SNP-graphene interface; **c**, **d** (i) F-actin (green), (ii) α-tubulin (red), (iii) Nuclei (blue), and (iv) merge. The scale bars represent 40 μm. **e**–**g** Time-resolved spectral changes induced by ROS generated from the cells under three different cellular conditions, including normal cells (**e**), NaAsO_2_-exposed normal cells (**f**), and cancer cells (**g**). **h** Plots of time-resolved changes in the quenching dip at 550 nm induced by ROS in **e**–**g**. (i) Quantitative analysis for DCFDA intensity in fluorescence images of intracellular ROS in Additional file 1: Fig. S7 (n = 10 for each group). Statical analyses were performed using one-way ANOVA (** indicates *p* ≤ 0.01, *** represents *p* ≤ 0.001)

Before monitoring the ROS in living cells, cellular adhesion on the plasmonic SNP-graphene interface was examined using immunofluorescence staining for F-actin (green), α-tubulin (red), and nuclei (blue). Human dermal fibroblasts (HDF) and human melanoma cell lines (A375P) were seeded on the prepared SNP-graphene interface and on a glass slide (as a control) (Fig. 5a–d). Fluorescent images of F-actin and α-tubulin revealed that both cell types cultured on the SNP-graphene interface adhered well, as in the case of the controls. Noticeably, the plasmonic SNP-graphene interface showed a higher cell density than the control, consistent with the reported result showing that the graphene substrate promotes cell adhesion [43].

Having demonstrated that the graphene-covered SNP serves as an excellent probe for ultrasensitive detection of ROS and provides a comfortable interface to cells, we next monitored the ROS generated from living cells in real time. The reduced Cyt *c* was pre-dispersed in cell media, and Cyt *c*-mediated PRET signals were directly collected from the cells adhered to the plasmonic SNP-graphene interface. We investigated three cases: normal cells (i.e., HDF), oxidative stress-induced cells (i.e., NaAsO_2_-exposed HDF), and cancer cells (i.e., A375P), as displayed in Fig. 5e–g. The time-resolved spectra exhibited dynamic changes in the quenching dip over time. In the case of A375P, the change at 550 nm is more prominent than that of HDF (Fig. 5 h). This is consistent with the fact that cancer cells maintain a higher ROS level than normal cells, which is associated with higher metabolic activity [66], activation of oncogenes [67], and mitochondrial dysfunction [18].

In addition, the HDF exposed to NaAsO_2_ clearly shows that the change in the quenching dip at 550 nm is greater than that of the unexposed HDF. Within 10 min, ROS levels were measured to the ranges comparable to the 10 nM–10 µM H_2_O_2_ for the three cases, when estimated from the calibration curve. After 60 min, the extracellular ROS levels were more clearly distinguishable according to the cellular conditions, with ROS levels comparable to 1 mM, 100 mM, and over 100 mM H_2_O_2_ for HDF, NaAsO_2_-exposed HDF, and A375P, respectively. Through our real-time measurements, we observed that stressed cells released ROS more rapidly into the extracellular environment than normal cells. The observed difference in ROS release kinetics is consistent with the fluorescence intensity for intracellular ROS production measured after additional incubation with a ROS-indicating dye, 2,7-dichlorofluoroscein diacetate (DCFDA) (Fig. 5i and Additional file 1: Fig. S7).

## Conclusions

We demonstrated the in situ optical monitoring of ROS generated from living cells on the plasmonic NP-graphene interface based on the redox-active Cyt *c*-mediated PRET signal. First, the optimal conditions for collecting clear PRET signals were systemically investigated by tuning the position, width, and intensity of the scattering spectrum of the probes by changing the type of plasmonic NPs (i.e., SNP, GNP) and number of graphene layers (i.e., 0 to 4 layers). We observed enhanced Cyt *c*-mediated PRET signals by modulation of electron movement through the interface between the NP and graphene. Using the optimized probes, we detected H_2_O_2_, a representative ROS, at the femtomolar level, which is six orders of magnitude lower than that of the commercially available ROS assay kit (ca. 1 µM). In addition, we demonstrated that a plasmonic-graphene hybrid interface provides an optically excellent and comfortable surface for real-time monitoring of cellular ROS from living cells. The proposed graphene-covered tunable plasmonic interface has versatile applications for studying cellular stress and disease progression by monitoring ROS levels under various cellular conditions.

## Supplementary Information

**Additional file 1: Figure S1.** Simulation of scattering spectra according to the number of graphene layers on SNP. (a) 110 nm SNP. (b) 120 nm SNP. (i) Full spectrum. (ii-iv) Plots showing shifts of λ_max_ (ii), FWHM (iii), and scattering cross-section (σ_sc_) (iv).** Figure S2.** Simulation of scattering spectra according to the number of graphene layers on GNP. (a) 55 nm GNP. (b) 60 nm GNP. (i) Full spectrum. (ii–iv) Plots showing shifts of λ_max_ (ii), FWHM (iii), and scattering cross-section (σ_sc_) (iv). **Figure S3.** Representative TEM images of the used SNPs. The average size (for n = 40) was observed to be 101.6 ± 5.0 nm (mean ± SD, nm). Scale bars represent 25 nm. **Figure S4.** TEM images of the used GNPs. Average size (for n = 40) was 49.5 ± 2.6 nm. Scale bars represent 25 nm. **Figure S5.** Scattering properties of the plasmonic GNP-graphene interface. (a) Dark-field scattering images of the graphene covered-plasmonic GNP with increasing number of graphene layers. The scale bars represent 10 µm. (b) Corresponding scattering spectra measured for the GNPs with increasing number of graphene layers. (c) Plots for the shifts in terms of λ_max_ (i), FWHM (ii), and intensity (iii) with increasing the graphene layer on the GNP. **Figure S6.** Changes in photoluminescence (PL) intensities of graphene-covered NPs. (a) SNP. (b) GNP. (i) Schematic diagram, (ii) PL spectrum, and (iii) Plot for the change in PL intensity of NP at 550 nm in the presence of graphene layer. **Figure S7.** Fluorescence images of intracellular ROS in cells. (a) HDF, (b) NaAsO2-exposed HDF, and (c) A375P. The green fluorescence indicates intracellular ROS visualized by staining with a ROS indicating dye, 2,7-dichlorofluoroscein diacetate (DCFDA). The scale bars represent 50 µm.

## Data Availability

The datasets used and/or analyzed during the current study are available from the corresponding author on reasonable request.
