# Supplementary material for: Ultrasensitive and real-time optical detection of cellular oxidative stress using graphene-covered tunable plasmonic interfaces
Source: Nano Converg. 2022 May 23;9:23. doi: 10.1186/s40580-022-00315-9 (PMC9127018; doi:10.1186/s40580-022-00315-9)
Supplement: Supplementary file 1 — Additional file 1: Figure S1. Simulation of scattering spectra according to the number of graphene layers on SNP. (a) 110 nm SNP. (b) 120 nm SNP. (i) Full spectrum. (ii-iv) Plots showing shifts of λmax (ii), FWHM (iii), and scattering cross-section (σsc) (iv). Figure S2. Simulation of scattering spectra according to the number of graphene layers on GNP. (a) 55 nm GNP. (b) 60 nm GNP. (i) Full spectrum. (ii–iv) Plots showing shifts of λmax (ii), FWHM (iii), and scattering cross-section (σsc) (iv). Figure S3. Representative TEM images of the used SNPs. The average size (for n = 40) was observed to be 101.6 ± 5.0 nm (mean ± SD, nm). Scale bars represent 25 nm. Figure S4. TEM images of the used GNPs. Average size (for n = 40) was 49.5 ± 2.6 nm. Scale bars represent 25 nm. Figure S5. Scattering properties of the plasmonic GNP-graphene interface. (a) Dark-field scattering images of the graphene covered-plasmonic GNP with increasing number of graphene layers. The scale bars represent 10 µm. (b) Corresponding scattering spectra measured for the GNPs with increasing number of graphene layers. (c) Plots for the shifts in terms of λmax (i), FWHM (ii), and intensity (iii) with increasing the graphene layer on the GNP. Figure S6. Changes in photoluminescence (PL) intensities of graphene-covered NPs. (a) SNP. (b) GNP. (i) Schematic diagram, (ii) PL spectrum, and (iii) Plot for the change in PL intensity of NP at 550 nm in the presence of graphene layer. Figure S7. Fluorescence images of intracellular ROS in cells. (a) HDF, (b) NaAsO2-exposed HDF, and (c) A375P. The green fluorescence indicates intracellular ROS visualized by staining with a ROS indicating dye, 2,7-dichlorofluoroscein diacetate (DCFDA). The scale bars represent 50 µm. [file 40580_2022_315_MOESM1_ESM.pdf]

Supplementary Information

Ultrasensitive and Real-time Optical Detection of Cellular Oxidative Stress Using Graphene-covered Tunable Plasmonic Interfaces

Hakchun Kim[1,†], Hyun Ji An[1,†], Junhee Park[1], Yohan Lee[2], Min Seob Kim[3], Seungki Lee[1], Nam Dong Kim[3], Jihwan Song[2], and Inhee Choi[1,*]

[1]Department of Life Science, University of Seoul, Seoul 02054, Republic of Korea

[2]Department of Mechanical Engineering, Hanbat National University, Daejeon 34158, Republic of Korea

[3]Institute of Advanced Composite Materials, Korea Institute of Science and Technology, Bongdong-eup, Wanju-gun, Jeollabuk-do 55324, Republic of Korea

[†]These authors contributed equally to this work

*Corresponding author: Inhee Choi

Tel.: +82-2-6490-2675

E-mail address: inheechoi1@uos.ac.kr

*Corresponding author: Jihwan Song

Tel.: +82-42-821-1084

E-mail address: jsong@hanbat.ac.kr

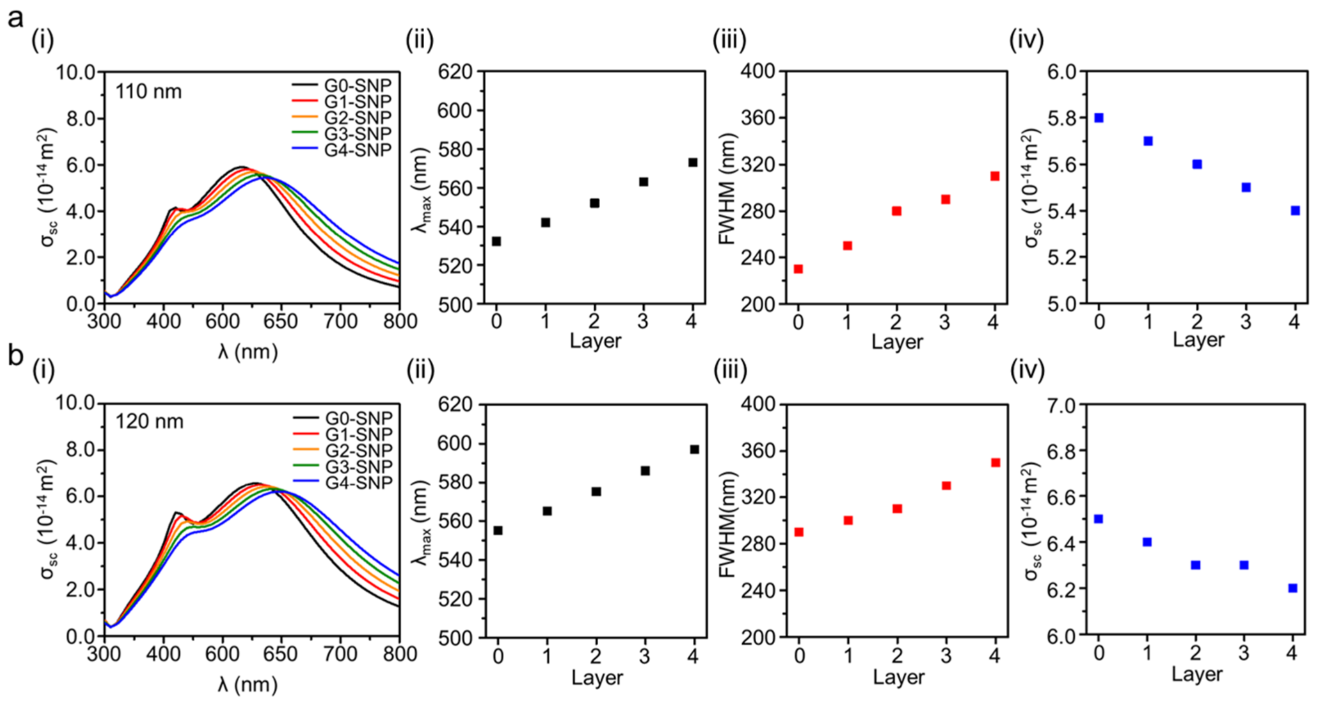

Fig. S1. Simulation of scattering spectra according to the number of graphene layers on SNP. (a) 110 nm SNP. (b) 120 nm SNP. (i) Full spectrum. (ii-iv) Plots showing shifts of λ_{max} (ii), FWHM (iii), and scattering cross-section (σ_{sc}) (iv).

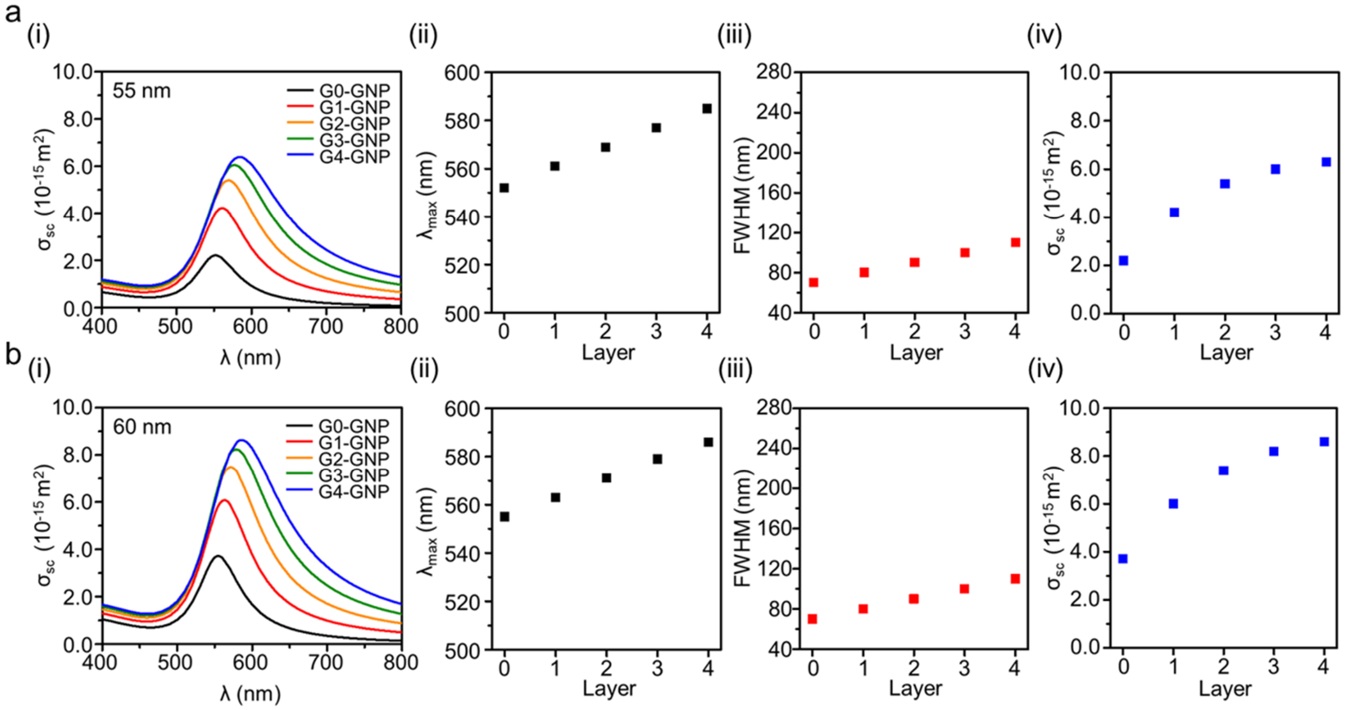

Fig. S2. Simulation of scattering spectra according to the number of graphene layers on GNP. (a) 55 nm GNP. (b) 60 nm GNP. (i) Full spectrum. (ii-iv) Plots showing shifts of λ_{max} (ii), FWHM (iii), and scattering cross-section (σ_{sc}) (iv).

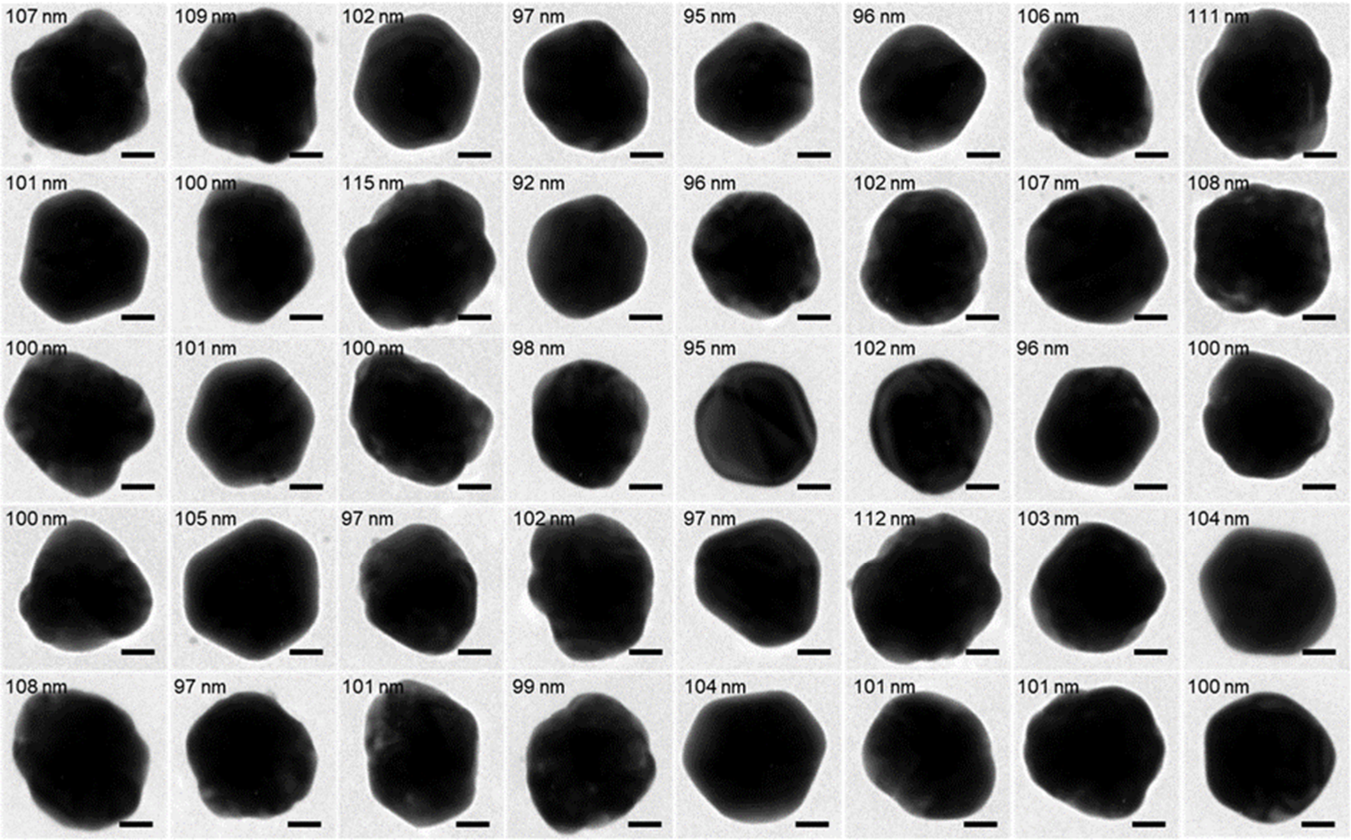

Fig. S3. Representative TEM images of the used SNPs. The average size (for n=40) was observed to be 101.6 ± 5.0 nm (mean ± SD, nm). Scale bars represent 25 nm.

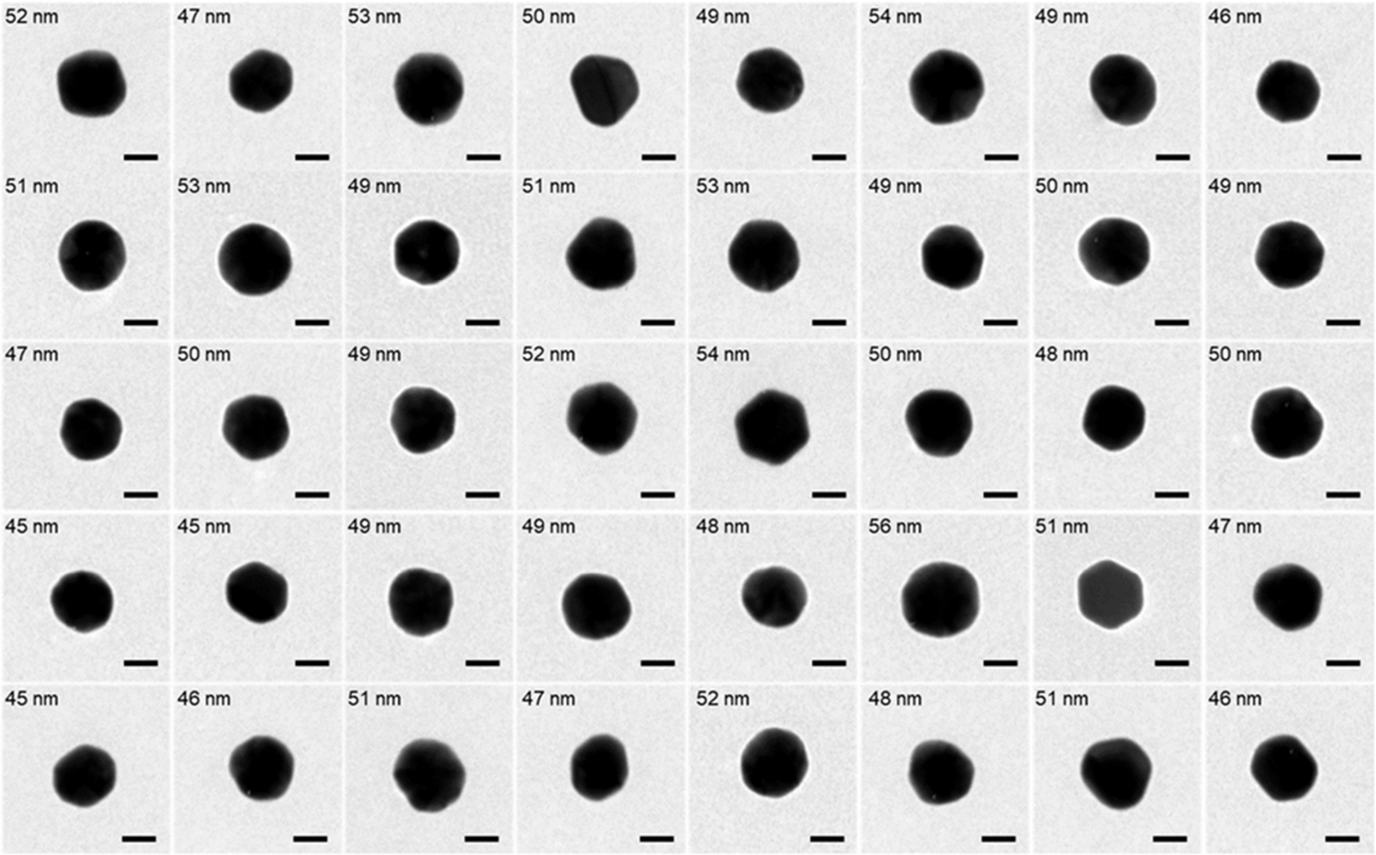

Fig. S4. TEM images of the used GNPs. Average size (for n=40) was 49.5 ± 2.6 nm. Scale bars represent 25 nm.

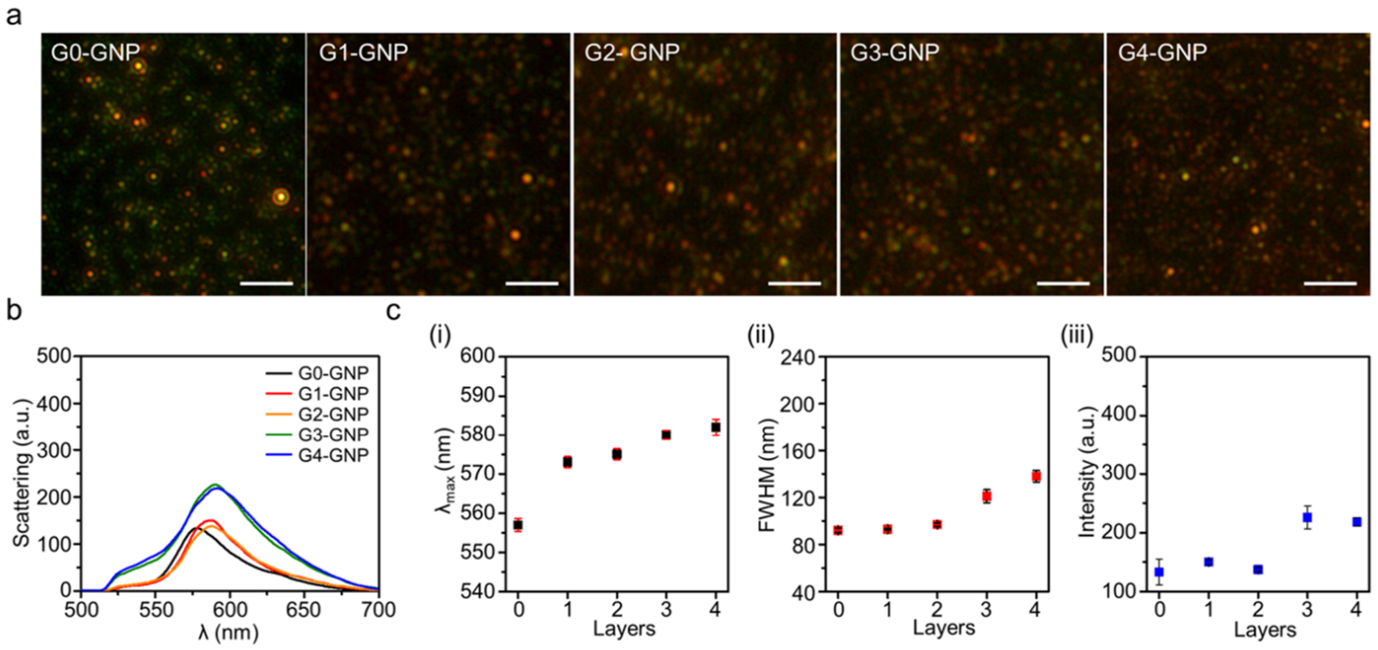

Fig. S5. Scattering properties of the plasmonic GNP-graphene interface. (a) Dark-field scattering images of the graphene covered-plasmonic GNP with increasing number of graphene layers. The scale bars represent 10 µm. (b) Corresponding scattering spectra measured for the GNPs with increasing number of graphene layers. (c) Plots for the shifts in terms of λ_{max} (i), FWHM (ii), and intensity (iii) with increasing the graphene layer on the GNP.

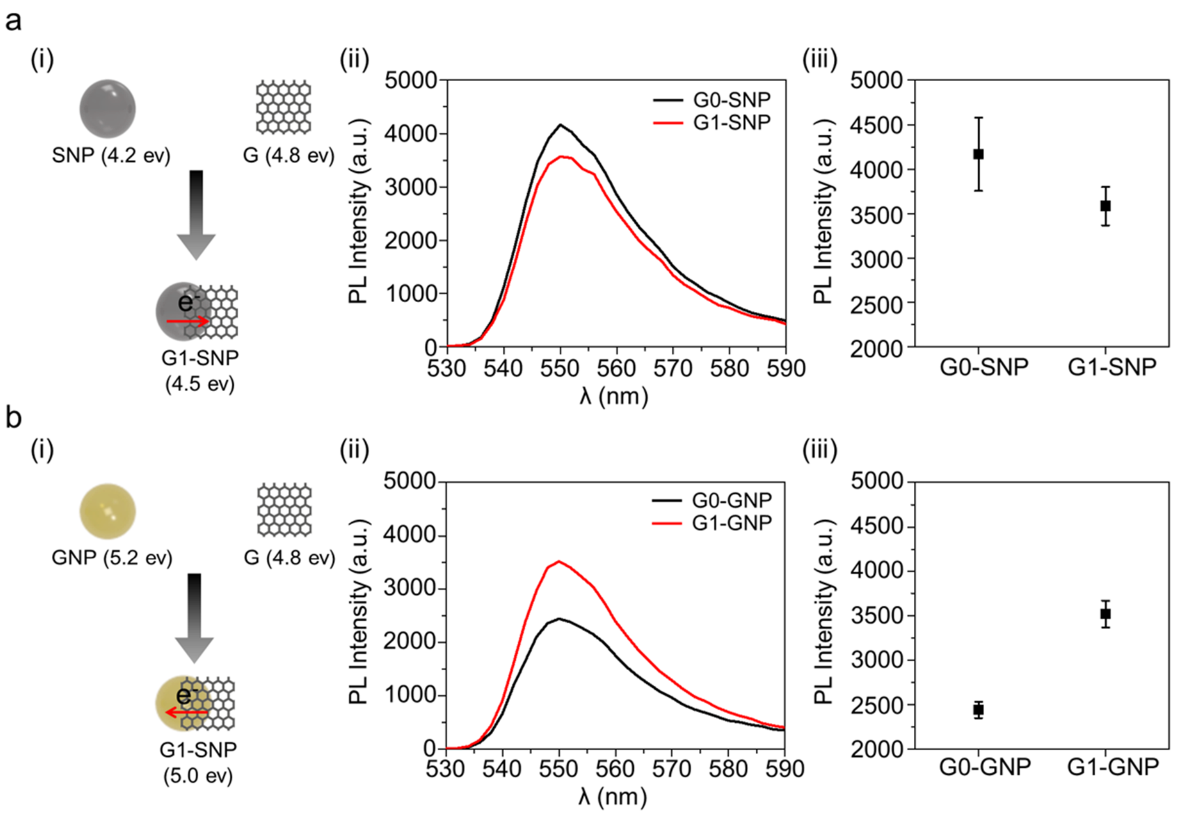

Fig. S6. Changes in photoluminescence (PL) intensities of graphene-covered NPs. (a) SNP. (b) GNP. (i) Schematic diagram, (ii) PL spectrum, and (iii) Plot for the change in PL intensity of NP at 550 nm in the presence of graphene layer.

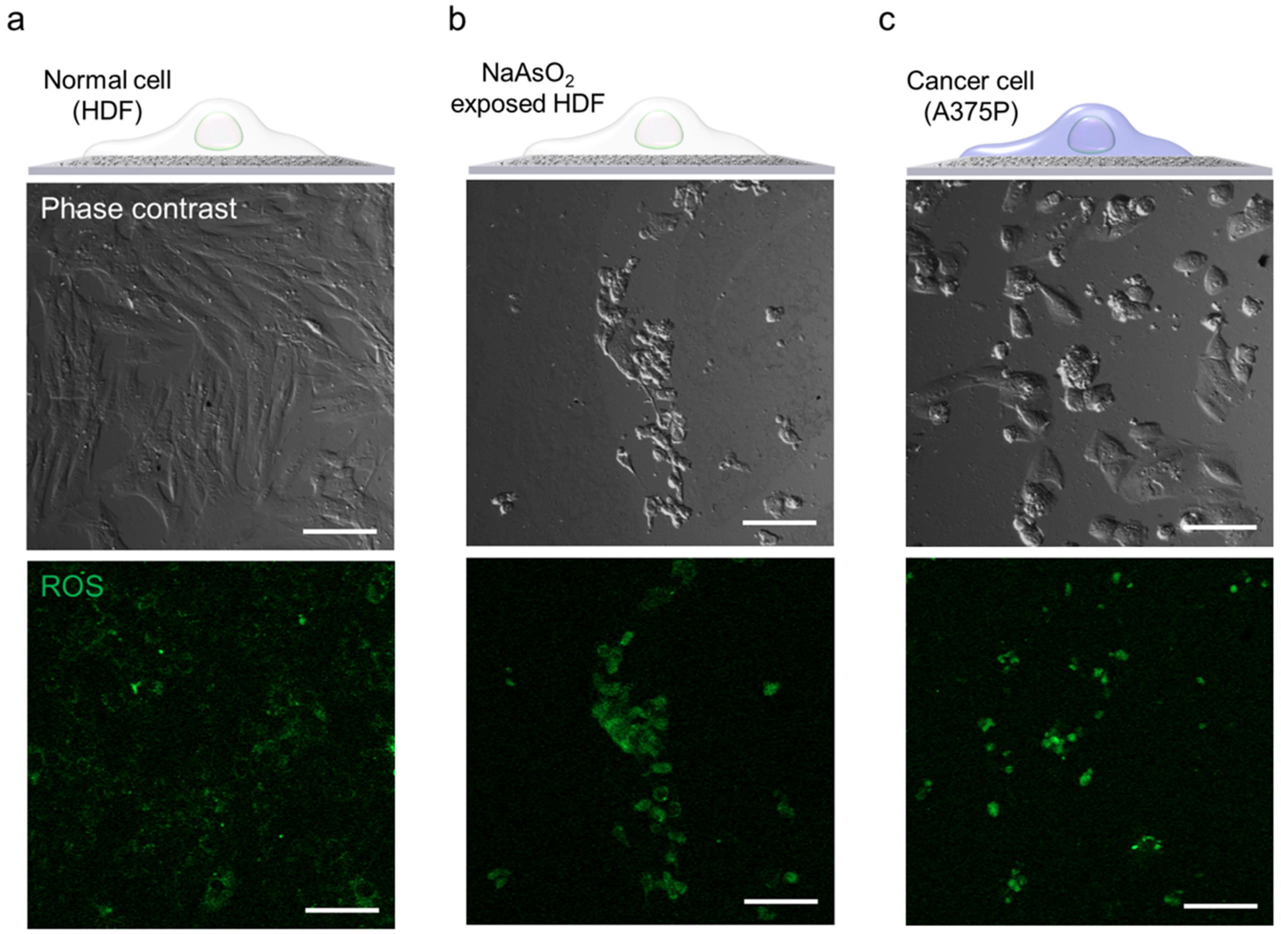

Fig. S7. Fluorescence images of intracellular ROS in cells. (a) HDF, (b) $NaAsO_2$-exposed HDF, and (c) A375P. The green fluorescence indicates intracellular ROS visualized by staining with a ROS indicating dye, 2,7-dichlorofluoroscein diacetate (DCFDA). The scale bars represent 50 μm.
